# Emotional Dysregulation in Preschoolers with Autism Spectrum Disorder—A Sample of Romanian Children

**DOI:** 10.3390/ijerph182010683

**Published:** 2021-10-12

**Authors:** Cristina Costescu, Mălina Șogor, Serge Thill, Adrian Roșan

**Affiliations:** 1 Special Education Department, Faculty of Psychology and Educational Sciences, Babes-Bolyai University, 400029 Cluj-Napoca, Romania; sogor.malina@gmail.com (M.Ș.); rosanadrianmarian@gmail.com (A.R.); 2 Donders Institute for Brain, Cognition, and Behaviour, Radboud University Nijmegen, 6525 GD Nijmegen, The Netherlands; s.thill@donders.ru.nl

**Keywords:** emotional dysregulation, autism spectrum disorders, physiological data, functional communication training

## Abstract

Emotional dysregulation problems seem to affect more than 80% of people with autism spectrum disorder (ASD) and may include irritability, aggressive behaviors, self-injury, and anxiety. Even though these types of problems are very common and affect the well-being of individuals with ASD, there are no objective assessment tools developed for this population and there are only a few intervention techniques meant to address these symptoms. This study investigates the feasibility of using off-the-shelf wearable devices to accurately measure heart rate, which has been associated with emotional dysregulation, and to test the effectiveness of functional communication training in reducing the emotional outburst in preschoolers with ASD. We used a single-case experiment design with three preschoolers with ASD to test if the duration of the emotional outburst and the elevated heart rate levels can be reduced by using functional communication training. Our results show that for two of the participants, the intervention was effective in reducing the duration of behaviors associated with emotional outburst, and that there were significant differences between baseline and intervention phase in terms of heart rate levels. However, our results are inconclusive regarding the association between elevated heart rates and the occurrence of the emotional outburst. Nevertheless, more research is needed to investigate the use of off-the-shelf wearable devices in predicting challenging behaviors in children with ASD.

## 1. Introduction

Autism spectrum disorder (ASD) represent a range of neurodevelopmental disorders characterized by deficits in social interactions and repetitive behaviors, stereotype behaviors and restricted interests [1], and are estimated to affect 13 to 29 per 1000 children aged 8 years old [2]. Furthermore, 80% of people with ASD have associated emotional regulation (ER) problems, which may include irritability, aggression, self-injury, anxiety, depression or impulsivity [3,4]. According to Thompson, ER represents “extrinsic and intrinsic processes responsible for monitoring, evaluating and modifying emotional reactions, especially their intensive and temporal features, to accomplish one’s goals” [5] (pp. 27–28). In addition, he outlines some possible methods that can help in regulating emotions: a. neurophysiological response—a brain-mediated somatic response; b. attentional processes—concentrating attention toward a stimulus in order to avoid focusing on the negative feelings; c. attributions—reinterpreting the situation in a way that can alter the emotional reaction; d. using a coping strategy—for example, discussing the situation with somebody; e. exposure to environment—when one context may help the person improve their emotional state; f. behavioral response—when the person engages in a pleasant activity in a deliberate manner.

In addition to the core features of ASD, recent studies have shown that children with ASD may also experience low frustration tolerance, irritability, inconsolable tantrums and aggressive behaviors, which are associated with ER problems [6,7,8,9]. When considering emotional dysregulation in children with ASD, it is important to analyze both their heightened levels of emotional reactivity and their ER strategies [10]. Both emotional reactivity, which represents the initial strength, intensity, speed and duration of the emotional response, and the use of adaptive ER strategies, such as attentional strategies, communication, or self-comforting behaviors, may impact the children’s behaviors.

There are several factors that may contribute to emotional dysregulation in ASD: Firstly, there could be neurobiological abnormalities, such as abnormal functioning and connectivity of the amygdala/prefrontal cortex, which may also influence the physiological activity and lead to hyperarousal. Secondly, there could be difficulties in the processing the social information; for example, atypical ways of perceiving environmental stimuli, or social cues [11,12]. Cognitive factors and executive dysfunction could also lead to emotional dysregulation in ASD—for example, poor cognitive flexibility, low planning abilities, difficulties in perspective taking or problem solving—and these factors could also be linked with less organized feelings and difficulties in accomplishing their goal-directed emotions [13]. Another possible factor that may influence emotional dysregulation in children with ASD could be the higher rates of negative affect, namely seeming to show greater or more intensive levels of negative emotions, which leads to psychiatric disorders, primarily anxiety and depression [14].

The capacity to regulate emotions is associated with proper social functioning and well-being [15] and with adaptation at school entry and social-communication [16,17]. Ineffective ER strategies and low self-control were associated with poor quality of life and mental health [18] and these types of maladaptive strategies were found in both young and older children with ASD, as well as in adolescents and adults [19,20,21]. Emotional dysregulation is associated with poor functioning in multiple domains; it can amplify the social deficits and can interfere with social and communication gains through behavioral therapy if not addressed [22]. Moreover, emotional dysregulation is associated with more symptoms of depression, anxiety, and externalization behaviors [23,24] and represents a critical barrier that interferes with success in terms of school performance and integration [25,26]. Difficulties with emotional regulation may be considered as a common mechanism that can explain these challenges [20]; more specifically, these difficulties relate to the evaluation and modification of emotional reactions, including their intensity and duration, in order to facilitate goal-directed behavior [15].

When investigating ER abilities in children with ASD, practitioners and researchers rely on measurements that were developed for typical developing (TD) children, and which were not adapted for children with ASD [27]. Therefore, researchers and practitioners face different challenges in measuring ER abilities; for example, one of the major problems is the verbal understanding of children with ASD and their capacity to express their feelings. Another possibility is to use questionaries that are completed by caregivers or teachers; however, these different ways of measuring ER skills may capture different aspects of the same construct [28]. The observation methods that include identifying children’s facial expressions or disappointment paradigm [29] may also reveal limited knowledge due to the abnormal use of nonverbal communication of children with ASD. Therefore, more objective measurement methods are required.

The emotion experiences are similar in the way they are associated with varying physiological arousal in typical developing children as well in children with ASD [30]. When interacting with different people in different contexts that increase psychological stress or negative emotions, the activity of the sympathetic nervous system (SNS) produces physiological arousal to help the child in adapting to the situation/challenge [31]. ER is strongly related to individual’s ability to adjust their physiological arousal [32]. This state of arousal can be identified through an increased pulse or heart.

More than two decades ago, the first study was published and described that physiological dysregulation may have a causal relation to children’s challenging behaviors [33]. Since then, several studies have established the connection between heart rate and emotional dysregulation in different samples; for example, children at-risk for externalizing disorders [34], children with conduct problems [35] and children with reactive aggression [36]. Therefore, heart rate may represent an objective measurement of emotional dysregulation and can also be considered a good factor in predicting the occurrence of challenging behaviors in children with ASD and other disorders. Two single-case studies have shown that elevated heart rates may be observed prior to disruptive behavior episodes in children with ASD. In those studies, heart rate measurements were investigated in the 5-seconds [37] or 30-s interval preceding the onset of challenging behaviors [38]. However, these short-time intervals may not inform and allow the intervention technique to be applied in real time. Therefore, analysis of a longer time window is needed. In addition, most of the studies were conducted with youth or older children, and it remains unclear how this relationship between heart rate and emotional dysregulation functions in preschoolers. Regarding ER strategies in Romanian children, a recent study conducted in this field [39] showed that adaptive strategies were more frequently used by typically developing children compared to children with ADHD or children with intellectual disabilities. The maladaptive ER strategies were associated with a high level of affective problems, anxiety, oppositional-defiant, and conduct problems in children in the ADHD group.

Among the difficulties encountered in the assessment process, treatment developers have also neglected the topic of ER, particularly since the studies focused more on behavioral and cognitive-behavioral therapy. Moreover, the existing interventions target mostly anxiety symptoms and are more focused on problem-specific techniques and their effectiveness on irritability, emotional outburst and challenging behaviors is not sufficiently investigated. The classical intervention techniques, such as reappraisal (very often targeted in cognitive-behavioral therapy) may be difficult to employ during the emotional outburst of children with ASD, mostly because of high emotional distress during those episodes. The techniques that are cognitively focused frequently go beyond the developmental capabilities of preschool-age children [40].

There is one study that reported promising results in a small sample of children with ASD aged 6 to 8 years old [41] when addressing ER in a modified cognitive-behavioral program. However, the outcomes were assessed using one-tailed comparison measured with child responses to vignettes and parent reports. Another study [42] investigated the feasibility of a manualized cognitive-behavioral program for improving ER in youth with ASD aged 8 to 12 years old. Their results showed that children showed less lability and negativity, less internalizing and behavior symptoms, and more adaptive behaviors. Conner et al. [43] implemented *Emotional Awareness and Skills Enhancement*, a program that is based on mindfulness and was piloted in two studies with 20 participants with ASD aged 12 to 17. Their outcome data support program feasibility and acceptability to participants, as well as demonstrating significant improvements in ER impairments and related concerns.

The purpose of our study is to assess the feasibility of a new objective measurement in the case of ER in children with ASD and to identify if the occurrence of emotional outbursts can be associated with elevated heart rate levels; it also aims to test if functional communication training can be effective in reducing emotional outburst in preschoolers with ASD. Therefore, our first hypothesis is that elevated heart rate levels are positively associated with the occurrence of the challenging behaviors connected with emotional outburst, our second hypothesis is that children with ASD will have a shorter duration of their emotional outburst in the intervention phase compared to baseline, and our third hypothesis is that is that children with ASD will have more elevated heart rate levels at the baseline phase compared to the intervention phase.

Functional communication training (FCT), the intervention that we propose to be tested in this study, as defined by Carr and Durrand [44], consists of assessing the behaviors via one or several functional assessments and teaching the child alternative behaviors as a communicative response [45]. FCT for people with ASD has strong empirical support in reducing challenging behaviors [46,47,48,49,50]; however, from our knowledge, its effectiveness in training adaptative ER strategies and reducing emotional outbursts has limited evidence.

## 2. Materials and Methods

### 2.1. Participants

Three participants located in Romania met the following inclusion criteria and participated in our study: (a). confirmed ASD diagnosis from an available clinical psychologist report and/or the Autism Diagnostic Observation Schedule [51]; (b). aged between of 3 and 5 years old; (c). difficulties in using ER adaptive strategies, i.e., frequent emotional outbursts; (d). demonstrated willingness to attend research and therapy sessions. The study was carried out according to the law concerning the conduct of psychological studies, including abidance by international ethical standards foreseen in the updated Helsinki Declaration of Human Rights. We obtained the approval of the faculty ethics committee to conduct the study under the code Research Ethical Approval/8 November 2020. Data were used in a manner that respected the regulation regarding the privacy of the subjects and the protection of their identities, and informed consent was obtained for each participant.

To assess their ER strategies, we used the Emotion Dysregulation Inventory [52,53]. The EDI is a caregiver report that identifies observable signs of poor emotional regulation or emotional dysregulation over the last 7 days, which are rated using a five-point Likert scale from “not at all” to “very severe”. The instrument has two parts, one part that measures Reactivity, such as rapidly escalating, intense and poorly regulated negative affect characterized by anger and irritability, and has a 24-item bank, and the other part measures Dysphoria, such as sadness, unease, low motivation and anhedonia, and this scale has 6 items. For the Reactivity Scale, a score above 25 is considered clinically elevated. Cronbach’s alpha internal consistency was 0.97 for Reactivity, and 0.92 and 0.90 for Dysphoria, according to the study conducted by Mazefsky, Yu, White, Siegel, and Pilkonis [53].

### 2.2. Design

The research design chosen for this study was the single case AB experimental design with inter-subject replications. We decided to implement this type of design because it has some characteristics that are common to classical experimental research, which compares control groups with experimental groups. For example, it allowed us to control the independent variable, use standardized measurement for the dependent variable and ensure the integrity of the independent variable [54]. Even if is a basic design, this type of design is commonly used in clinical settings and has a great utility in measuring the magnitude of the outcome change [55,56].

### 2.3. Procedure

All of the therapy sessions for all of the participants were implemented by a clinical psychologist, who was trained by the experimenter (second author), in a therapy room at a service center for children with ASD in Romania. The experimenter, who is a specialist in assessment and interventions for children with neurodevelopmental disorders, observed all assessments sessions and therapy sessions and collected the data without intervening in the therapeutic setting. Firstly, there was a habituation phase, in which the experimenter was introduced to the child; secondly, the baseline phase was implemented; and thirdly, the intervention sessions were conducted.

Besides the above-mentioned instruments that were used for initial assessment, we also used a behavioral grid that contained the description of each of the emotional outburst and associated behaviors of the child, the trigger, the duration, and the latency of the behaviors. Moreover, for the physiological data collection, we used the Xiaomi Mi Band 4 Fitness Bracelet for measuring the pulse of the child. Previous studies showed that wrist-worn devices adequately measure heart rate in laboratory-based activities [57]. We measured the pulse of the child minute-by-minute and for every emotional outburst that happened in the therapy session for 5 sessions in the baseline phase and for 5 sessions in the intervention phase. To establish whether it is possible to associate the appearance of the emotional outburst and the challenging behaviors, we also analyzed the pulse before the emotional outburst appeared. Therefore, each child had 25 measurements in the baseline phase and 25 measurements in intervention phase.

We followed the standard steps in the development functional communication training, and we firstly identified the challenging behaviors whose duration we chose to reduce; afterwards, we decided—together with the child’s therapist—which is the more appropriate way for the child to communicate his/her need. In the implementation phase, we systematically taught the child the new communication skill, meaning that every time the child went through an emotional outburst, he/she was directed/prompted to exercise the new communication skill. For all three children, we followed the same structure of the new communication skill; at the beginning, we encouraged the child to recognize the emotion (that they felt), to name the emotion and to use a functional request (by communicating it to the therapist). Each time the child used the new communication skill, we reinforced his/her behavior and we reminded the child to use the new skill whenever the difficult behavior appeared. The intervention was implemented every time that the pulse was increasing, which was the signal that an emotional outburst would follow, and with consideration of the triggers that were observed in baseline. Their adaptative ER strategy (i.e., naming how they feel and communicating functionally with their therapist) were verbally reinforced every time in the intervention phase.

### 2.4. Data Analysis

To test our hypothesis, we compared the duration of the emotional outburst and the heart rate that the three participants showed in the two phases of the experiment, baseline and intervention, using a non-parametric Wilcoxon test. This type of analysis allowed us to identify if the intervention was effective in reducing the emotional outburst and the behavior associated with that, namely the duration of the behavior and the heart rate intensity. We also reported the visual analysis of the graphs, considering the following indicators: trend of the data (tendency of the data—ascending or descending), magnitude of the data (numerical difference between values from phase A and B) and latency (time taken/number of sessions before the change occurred).

Inter-observer agreement data were collected for 30% of baseline sessions and 30% of intervention sessions across the sessions developed for this study. Inter-observer agreement data were collected for 40% of intervention sessions for treatment fidelity. Research assistants were provided with behavioral definitions and trained by the second author using sample videos until they could reach a minimum of 90% agreement across three consecutive sessions before beginning formal data collection. The first author randomly selected videos for inter-observer agreement data. To check the reliability of data scoring, Cohen’s Kappa Coefficient was used. The Kappa scores obtained in the test of the subset of trials for the children was 0.86, indicating a good reliability.

## 3. Results

To identify whether heart rate is an accurate measurement of the emotional outbursts, we measured the heart rate of the three participants in the study, on a minute-by-minute basis, during the baseline and intervention sessions. Detailed participant characteristics are given below.

*The first participant of the study is S.R.* She is a 4-year-old girl who was diagnosed with ASD. From the *social* point of view, S.R. looks at the faces of people around her and smiles. She also looks for the toys she intends to play with and she expresses her desires to play. She can take part in different activities that involve symbolic games; for example, she can pretend to feed or wash a doll. S.R. uses “please!” and “thank you!” in interactions with other children only when she is reminded to, and she offers a greeting when she sees a person with whom she is familiar. She can bring or take objects from another room or find a person in the next room. S.P. can name feelings such as sadness, happiness and love, but she cannot answer simple questions regarding her feelings when she is asked, for example, “How do you feel?”.

Regarding her *autonomy* she can eat by herself, using a spoon/fork and drink from a cup. She tries to help her parents with chores by doing a part of the necessary work, and she dresses alone with a shirt or undresses herself, brushes her teeth with help, in addition to washing and drying her hands. Considering her *motor skills*, she can rotate buttons, push on the doorknob, build a tower using cubes, turn the pages of a book one by one, use scissors, and make small balls from clay. She cannot fold paper; S.R. grabs the pen between the thumb and index finger, relying on the middle finger, but cannot unpack small objects. She can jump with both feet, go down the stairs with help, throw a ball to an adult from a distance, kick a ball that is not moving, hits nails with the hammer, walk backwards, jump forward, stand on one leg at a time for one second, walk on tiptoes, and kick a ball that comes toward her. She does not have the ability to pedal on a tricycle, run with coordinated movements and alternate her hands.

Regarding her *language* development, she uses simple two-word phrases, expressing her basic needs and possessions, and uses “no” in spontaneous dialogue. She answers to simple questions, such as “what are you doing?” and “where are you?”. S.R. recognizes the source of some familiar noises in her surroundings. She uses some nouns that define categories, she sometimes uses “I can/cannot” and some forms of the future tense, she can describe an object as “open” or “closed”, she can say her whole name upon request, and she can say what an ordinary object does. On the other hand, she has difficulties in using plurals and some irregular verbs in the past tense. She also seems to lack knowledge on the use of the definite and indefinite article with words of the masculine and feminine gender when speaking, she cannot answer common questions containing “how” or “when”, and she is not able to describe recent events. S.R. can name more geometrical shapes and colors, can name objects that are alike and different, can name body parts and can count to ten. She cannot draw a square via imitation, and is unable to add a hand or a foot to an unfinished drawing. 

Regarding her *emotional* development, she scored 41 points on the 24-item Reactivity Item Bank from the *Emotion Dysregulation Inventory* (*EDI*), which means that she has an elevated level of emotional dysregulation. She has explosive outbursts, her reactions are usually more severe than the situation calls for, she is easily frustrated and breaks down (crying, screaming) if told she cannot do something, has extreme or intense emotional reactions, her emotions can instantly increase from 0 to 100 in terms of intensity, and it is difficult to distract her if she is severely frustrated or upset. Regarding the 6-item bank for Dysphoria, S.P. has a score of 3 points, which correlates with a normal level of development. She does not refuse to leave the house or go to school, she participates in activities of her own volition, and it is not hard to calm her down when she is mad or upset.

To assess the disruptive behaviors, a functional assessment was implemented, assessing the contextual factors that led to the manifestation of that behavior. An observational grid was used, which recorded the duration and latency of the emotional outburst. After analyzing the functional assessment reports, we discovered that the contextual factors that triggered the disruptive behaviors were the use of the repetitive prompts by the therapist and her attempts to redirect the child into another activity because the participant had a low frustration tolerance

*The second participant of the study is I.M.* She is a 5-year-old girl who was diagnosed with ASD. From a social point of view, she has good eye contact, but she does not smile as a response during communication. She can take part in different activities involving symbolic games, and she can express her wishes and preferred activities. In interactions, she usually says “please!” and “thank you!” when she is reminded to, offers a greeting when she sees a person with whom she is familiar, and says “Bye” when prompted. She can bring or take objects or find a person in the next room. Although she partially understands and can name feelings such as sadness, happiness and love, she cannot express her feelings by answering the question “how do you feel?”.

With regard to her *autonomy*, she can eat alone, using a spoon/fork and drink from a cup. She tries to help her parents with chores by doing a part of the necessary work, and she undresses alone, but she needs help with dressing. This is also due to her *fine motor skill* difficulties, because she can push small buttons, push on the doorknob, build a tower using cubes, turn the pages of a book one by one, use scissors, and make small balls of playdough, but she is not able to close staples or to open buttons, and she has also trouble in holding a pencil using the pincer grasp. Concerning her *gross motor skills*, I.M. jumps with both feet, and she is able to climb stairs without help. She cannot throw a ball to an adult from a distance of over 1.5 m, make a tumble, run 10 steps with coordinated movements or alternate her hands; however, she is able to peddle on a tricycle.

Regarding her *language* development, she uses simple two-word phrases, composed of either a verb and a noun or an adjective and a noun, to express her needs or to describe the things and activities around her. She has also expressed possession (“my ball”) and uses “no” in spontaneous speech, she does not give an answer to the question “what are you doing?” for spontaneous activities, and she does not respond to the question “where are you?”. I.M. recognizes the source of some familiar noises in her surroundings, she is able to tell people if she is a boy or a girl, she uses some nouns that define categories, she sometimes uses “I can/cannot” and some forms of the future tense, she is able to name several opposite notions, she can say what an ordinary object does, she can express a wish by saying, “I want”, and she uses some nouns. She cannot answer simple social questions, such as “what is your name?” or “how old are you?”, and she cannot describe recent events or tell whether an event happened before or after another event. She can name geometrical shapes and colors, as well as objects that are alike and different, and she can count to ten. She cannot follow a given pattern of colors or shapes or follow more than a few lines from a story. 

Regarding her *emotional* development, she scored 39 points on the 24-item Reactivity Item Bank, meaning that she has elevated symptoms of emotional dysregulation. She has explosive outbursts, her reactions are usually more severe than the situation calls for, she is easily frustrated, and she has extreme or intense emotional reactions with a moderate degree of frequency. She breaks down (crying or screaming) if told she cannot do something, and her emotions instantly increase from 0 to 100, in terms of intensity, with moderate frequency. Moreover, she is difficult to distract if she is frustrated or upset. Regarding the 6-item bank for Dysphoria, the participant has a score of 6 points. She is responsive to praise or good things happening, and she does not seem to be sad or unhappy. She appears uneasy throughout the day and refuses to leave the house, go to school or participate in activities unless forced in a moderate way.

To assess her disruptive behavior, a functional assessment was implemented, and we considered the contextual factors that led to the manifestation of the emotional outburst and disruptive behaviors. Data were collected for all the participant by using an observational grid that recorded the duration and latency of the behaviors. We discovered, after analyzing the behavioral grids, that the emotional outburst was triggered by the withdrawal of favorite toy that was used as reinforcement or by delays in giving the promised toy/object as a reinforcement.

*The third participant of the study is G.M.* She is a 3-year-old girl who was diagnosed with ASD. From a *social* point of view, she has only a moderate interest in interacting with other people, she offers greetings only when prompted, and she does not play with other children even if she is asked to. G.M. does not seem to understand emotions and she cooperates with the parents 50% of the time; she rarely stops when she told to or when she is in danger. She has a high level of hyperactivity, and she likes to explore new toys and surroundings; however, she has difficulties in playing with toys in a functional or symbolic way. 

Regarding her *autonomy*, she can unbutton a shirt by herself, she can put on a coat but cannot zip the zipper without help, and she undresses by herself, but cannot take off her shoes without help. She can use a fork/spoon, can drink from a cup and eat alone, and brushes her teeth with help. She cannot put on a shirt or pants by herself and, most of the time, she needs to be reminded to use the bathroom to avoid accidents. Her autonomy is also limited by her *fine motor skills*, and she has difficulties in grasping small objects, grabbing a pencil between the thumb and the index finger, folding paper, or using scissors. Regarding her gross motor skills, she can walk up the stairs alone, throw a ball forward, jump and kick the ball with the foot, jump with both feet at the same time, walk backwards and walk up and down the stairs with help, and push the doorknob.

Her *language* abilities are limited as well. She can give an answer to yes/no questions, and can name a few familiar objects, body parts and family members. She uses mostly one-word sentences to express her needs and is rarely able to combine gestures and words to communicate with others. Her language is non-intelligible to unfamiliar adults, and she cannot name actions conducted by herself or others. She has difficulties in associating objects with categories and she cannot follow instructions that require the understanding of locations.

Regarding her *emotional* development, she scored 29 points on the 24-item Reactivity Item Bank, meaning that she has elevated symptoms of emotional dysregulation. She seems to frequently experience anger or irritation, is easily frustrated, and seems to be on edge and have trouble calming herself down. She also has mood swings almost daily. Regarding the 6-item bank for Dysphoria, G.M. has a score of 6 points. She usually refuses to leave the house, go to school or participate in activities unless helped by the adults around her, and she appears to be uneasy throughout the day.

To assess her disruptive behavior, a functional assessment was implemented, and we considered the contextual factors that led to the manifestation of the emotional outburst and disruptive behaviors After using the observational grid, the analysis revealed that the contextual factors that triggered the disruptive behaviors were the use of the repetitive prompt by the therapist and his attempts to redirect the child into another activity; this may be due her low frustration tolerance.

**Figure 1:**
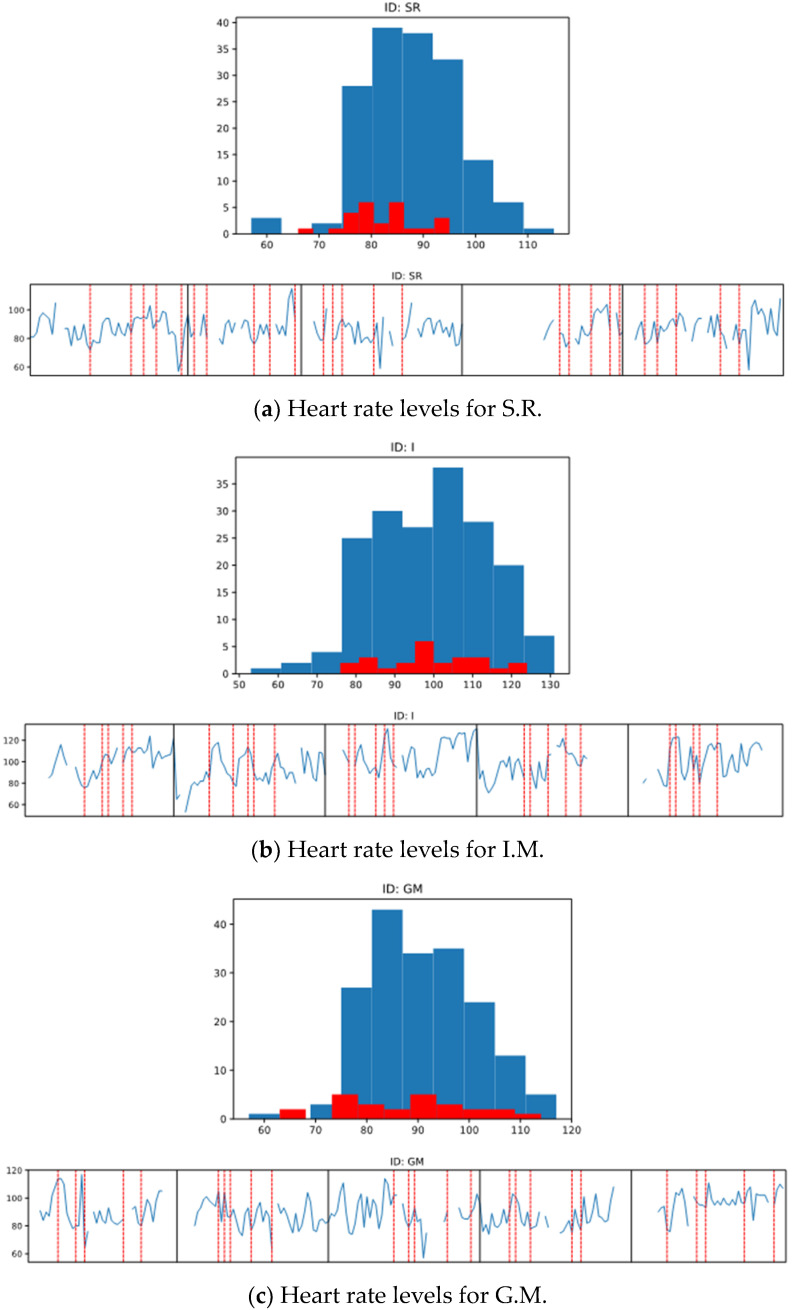
Heart rate levels for the participants and overlap between the emotional outburst and the intensity of the heart rate.

Our results show that for one of the participants, the heart rate increased during the emotional outbursts compared to other periods of therapy (Figure 1b), and the mean was 99.6 (SD = 12.74) during the emotional outburst compared to 99.0 (SD = 14.90) during other moments in therapy. For the other two participants (Figure 1a,c), the mean of the heart rate during the emotional outbursts was lower than the mean of the heart rate from other therapy moments. For S.R., the mean of heart rate intensity during the emotional outbursts was 82.16 (SD = 6.90) and during other moments of therapy the mean was 87.62 (SD = 9.01). For G.M., the mean of heart rate intensity during the emotional outbursts was 87.64 (SD = 12.48) and during other moments of therapy mean was 90.48 (SD = 10.18). In addition, to identify whether we could anticipate the occurrence of the emotional bursts, we looked at the heart rate value before the appearance of the behaviors, but the pulse seemed to increase very fast, and the data that were collected and analyzed minute-by- minute could not anticipate the occurrence of the behaviors. Nevertheless, the graphic analysis revealed that emotional outbursts were associated with elevated hear rate values for all the three participants, but apparently, there were also other moments in the therapy sessions that increased the heart rate levels; however, these were not identified in our study. 

### 3.1. Effectiveness of the Functional Training Intervention in Reducing the Duration of the Behaviors

To test the effectiveness of the functional training intervention for reducing the duration of the behaviors associated with the emotional outburst and the heart rate level, we conducted a statistical analysis. Our results show that all three participants registered a decrease in the duration of the behaviors associated with the emotional outbursts, but the decrease was significant from a statistical point of view in only two cases. 

#### 3.1.1. Participant S.R.

**Figure 2:**
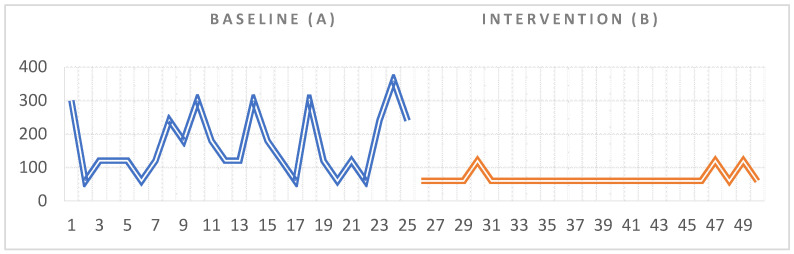
Visual representation of the data collected for S.R during the baseline and intervention phase.

Statistical data indicated that there was a significant difference between the duration of the behaviors associated with the emotional outbursts recorded at baseline (A) and the duration of the same behaviors recorded in the intervention phase (B) (Z = 3.78, *p* < 0.001) for S.R (see Table 1 for means and standard deviations). Regarding the visual analysis of the graph, we could not observe any trend in the data, neither in the baseline phase (A) nor in the intervention phase (Figure 2). Regarding the second indicator of the visual analysis (magnitude), we could observe a significant difference between the two phases as the duration of the behaviors decreased from 3930 s (65.5 min), recorded in the 25 measurements in A, to 1680 s (28 min) recorded in the 25 measurements in B, with the overall difference being 2250 s (37.5 min). We also identified that the effects of the intervention appeared after the first session for S.R.

#### 3.1.2. Participant I.M.

**Figure 3:**
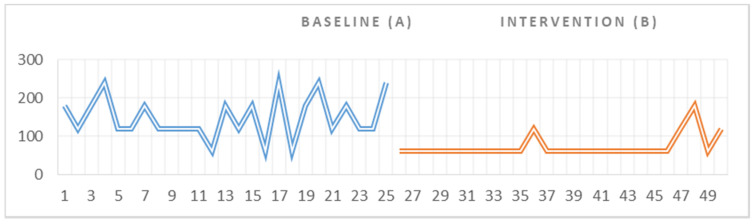
Visual representation of the data collected for I.M. during the baseline and intervention phase.

Statistical data indicated that there was a significant difference between the duration of the behaviors associated with the emotional outbursts recorded at baseline (A) and the duration of the same behaviors recorded in the intervention phase (B) (Z = 3.89, *p* < 0.001) for the second participant. With regard to the visual analysis of the graph, we could not observe any trend in the data, neither in the baseline phase (A) nor in the intervention phase (Figure 3). Regarding the magnitude of change, we could observe a significant difference between the two phases; the duration of a behaviors associated with the emotional outburst decreased from 3721 s (62 min), recorded within the 25 measurements in A, to 1800 s (30 min), recorded within the 25 measurements in B, and thus, a major difference of 1921 s (32 min) was detected. Similarly to first participant of the study, we identified a change in the durations of the negative behaviors even from the first session of intervention.

#### 3.1.3. Participant G.M.

**Figure 4:**
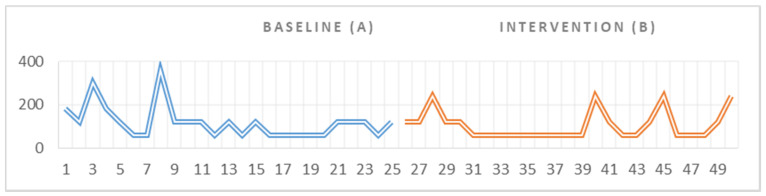
Visual representation of the data collected for G.M. during the baseline and intervention phase.

For our third participant, the data showed that there were no significant differences between the duration of the behaviors associated with the emotional outbursts recorded at baseline (A) and the duration of the same behaviors recorded in the intervention phase (B) (Z = 0.525 *p* = 600). Following the visual analysis of the graph, we could observe a downward trend in the data within phase A, indicating a decrease in tantrum duration in the baseline setting. We could not identify any trend in the visual analysis of the graphic (Figure 4); however, there was a small decrease in the duration of the negative behaviors from 2940 s (49 min), recorded in the 25 measurements in A, to 2640 s (44 min), recorded in the 25 measurements in B, with a difference of 300 s (5 min).

### 3.2. Effectiveness of the Functional Training Intervention in Modifying the Heart Rate 

Regarding the analysis conducted to see if there were any changes in heart rate levels in the two phases of the study, baseline and intervention, significant differences were identified for two of the participants (see Table 2 for means and standard deviations).

Statistical analysis of data for S.R. indicated that there was a significant difference between the heart rate of the participant in phase A compared to the heart rate manifested by the participant in the phase B intervention (Z = 3.11, *p* = 0.002). We observed that the highest pulse manifested by S.R. in phase A during an emotional outburst was 95, while the highest pulse manifested by S.R. in phase B following the intervention was 89. In addition, similar results were identified for I.M., namely a significant difference between the participant’s heart rate in phase A compared to the heart rate exhibited by the participant in the phase B intervention (Z = 2.57, *p* = 0.001). Regarding our third participant, G.M., the statistical data indicated that there was no significant difference between the participant’s heart rate in phase A and the heart rate manifested by the participant in the phase B intervention (Z = 0.83, *p* = 0.404).

## 4. Discussion and Conclusions

Emotional outbursts are common problems associated with children and adults with ASD. The intensity, frequency and duration of the behaviors associated with the emotional outbursts vary a lot and children with ASD have difficulties in overcoming these problems. In therapy and assessment, the use of the standard functional analysis of the behaviors is essential for treatment planning; however, is not sufficient to have a clear, objective view of the problem in terms of helping to prevent the emotional outburst from occurring again. There are several studies showing the association between the occurrence of challenging behaviors and heart rate modifications [33,34,35,36,37,38]; therefore, we consider the use of off-the-shelf wearable devices for the monitoring of heart rate in order to predict emotional outbursts to be a novel idea that still needs to be investigated in further studies. However, there are some important aspects that need to be considered, such as the type of device used and its validity in terms of collecting accurate heart rate data, the age of the children included in the study, and the intervals of the heart rate measurements analyzed. Our results show that using off-the-shelf wearable devices for the monitoring of heart rate to predict the appearance of an emotional outburst is a feasible idea and that high intensity heart rate levels are associated with emotional outbursts. Even though our results are inconclusive, and our first hypotheses was rejected, there is a huge potential in using fitness bracelets for children with ASD, particularly in helping teachers and parents to identify, in a more accurate and objective way, when an emotional outburst is about to happen. Despite the challenges involved in measuring ER strategies in children with ASD, this field has a great potential to improve the understanding of challenging behaviors in ASD and to offer means of diverting their manifestation [58]. The understanding of the underlying mechanisms could also help in developing effective interventions to improve their emotional and behavioral problems. It is clear that there is a need to employ a multimethod approach in studying ER in children with ASD [13], especially because physiological data have the advantage being objective regardless of the children’s level of understanding, language or emotional development. One possible explanation for the discrepancies that we found between heart rate values during emotional outbursts and regular emotional states of the child would be that preschoolers may not yet have a stable system of emotional regulation strategies [59]. Studies show that elevated heart rate levels predict the occurrence of challenging behaviors more accurately when considering older children with ASD [60].

ER therapy, as defined and manualized by Mennin et al. [61], consists of 16 weekly sessions that combine cognitive and behavioral strategies, and target worry, rumination, avoidance and compulsive behaviors. The developed skills during the therapy sessions aim at helping the clients to identify and use alternative reactions, other than worry or avoidance, that they are used to. In the intervention that we proposed in this study, we upheld the main principals of ER therapy and we helped children to identify the arising of emotions and use adaptative ways of expressing their needs. We also used the FCT to improve their ER strategies because previous studies in the field showed that children with ASD use mostly maladaptive involuntary forms of ER, including remaining focused on the stressor or being unable to think or act [20]. By using FCT, we taught preschoolers with ASD more acceptable behaviors that serve the same function as their problem behavior. In addition, by adding the two more components, such as asking their therapist for what they need, and recognizing and naming the emotion they feel, helped children better understand their feelings and reactions. Therefore, our second hypothesis and third hypothesis were confirmed for two out of three participants, both of whom showed decreased challenging behaviors associated with the emotional outbursts, and their heart rate levels also decreased.

There are several limitations that should be considered when interpreting the results of our study. Even if the purpose of the study was to test the feasibility of using off-the-shelf wearable devices in the therapy of children with ASD, the data collection method may have influenced the data; for example, we considered only minute-by-minute heart rate levels, which can be a limiting factor. Moreover, the wearable device used to measure physiological has some limitations in terms of accurately depicting heart rate, and the small number of cases analyzed in this study should also be taken into account. Considering the above-mentioned limitations, we recommend that our results be interpreted with great caution. Future studies should analyze the heart rate levels at different intervals to achieve greater accuracy of measurement, and should also utilize larger sample sizes. We only analyzed 10 sessions, of 50 min each, as well as 5 sessions at baseline and 5 sessions at the intervention stage, to accurately predict or identify a pattern in the dataset; therefore, more data needed to be collected. Moreover, concerning the intervention that we applied, simple replacement of the challenging behavior with a form of adaptative communication requires closer attention to the consequences of the behaviors, the types of the responses taught and context in which they are being used.

In conclusion, taking into account previous studies involving Romanian children, which showed that children with neurodevelopmental disorders have difficulties in controlling their anger and adopting adaptive emotional regulation strategies, as well as the need to develop effective measurement and intervention techniques, our study attempted to identify and test the effectiveness of such instruments. Future studies should use wearable devices that offer reliable physiological internal state monitoring and may be suitable for people with ASD [58], and should test their effectiveness, not only as assessment instruments, but also as part of the intervention techniques that are used.

## Figures and Tables

**Table 1:** Means and standard deviations in Phase A and Phase B for all three participants.

	Phase A M (SD)	Phase B M (SD)
S.R.	168 (91.65)	67.20 (19.89)
I.M.	148.80 (55.09)	72 (30)
G.M.	117.60 (74.45)	105.60 (65.45)

**Table 2:** Means and standard deviations for heart rate measurements in Phase A and Phase B for all three participants.

	Phase A M (SD)	Phase B M (SD)
S.R.	82.32 (7.01)	77.28 (8.97)
I.M.	100.8 (12.31)	89.68 (12.88)
G.M.	87.64 (12.74)	91.64 (14.59)
